# Baicalin and probenecid protect against *Glaesserella parasuis* challenge in a piglet model

**DOI:** 10.1186/s13567-024-01352-4

**Published:** 2024-07-29

**Authors:** Shulin Fu, Siyu Liu, Jingyang Li, Qiaoli Dong, Yunjian Fu, Ronghui Luo, Yamin Sun, Xinyue Tian, Wei Liu, Bingbing Zong, Chun Ye, Qirong Lu, Yinsheng Qiu, Ling Guo

**Affiliations:** 1 Hubei Key Laboratory of Animal Nutrition and Feed Science, Wuhan Polytechnic University, Wuhan, 430023 China; 2 Hubei Collaborative Innovation Center for Animal Nutrition and Feed Safety, Wuhan, 430023 China

**Keywords:** baicalin, probenecid, *Glaesserella parasuis*, protection, piglets

## Abstract

*Glaesserella parasuis* (*G. parasuis*) induces vascular damage and systemic inflammation. However, the mechanism by which it causes vascular damage is currently unclear. Baicalin has important anti-inflammatory, antibacterial and immunomodulatory functions. In this study, we explored the ability of baicalin and probenecid to protect against *G. parasuis* challenge in a piglet model. Sixty piglets were randomly divided into a control group; an infection group; a probenecid group; and 25 mg/kg, 50 mg/kg and 100 mg/kg baicalin groups. The probenecid group and the 25 mg/kg, 50 mg/kg and 100 mg/kg baicalin groups were injected intramuscularly with 20 mg/kg body weight (BW) probenecid and 25 mg/kg BW, 50 mg/kg BW and 100 mg/kg BW baicalin, respectively. All piglets except those from the control group were injected intraperitoneally with 1 × 10^8^ CFU of *G. parasuis.* The control group was injected intraperitoneally with TSB. The results showed baicalin and probenecid protected piglets against *G. parasuis* challenge, improved body weight and decreased temperature changes in piglets. Baicalin and probenecid attenuated IL-1β, IL-10, IL-18, TNF-α and IFN-γ mRNA levels in the blood for 48 h, inhibited the production of the nucleosides ATP, ADP, AMP and UMP from 24 to 72 h, reduced Panx-1/P2Y6/P2X7 expression, weakened NF-kB, AP-1, NLRP3/Caspase-1 and ROCK/MLCK/MLC signalling activation, and upregulated VE-cadherin expression in the blood vessels of piglets challenged with *G. parasuis*. Baicalin and probenecid alleviated pathological tissue damage in piglets induced by *G. parasuis*. Our results might provide a promising strategy to control and treat *G. parasuis* infection in the clinical setting.

**Supplementary Information:**

The online version contains supplementary material available at 10.1186/s13567-024-01352-4.

## Introduction

*Glaesserella parasuis* (*G. parasuis*) is a common bacterial community in the upper respiratory tract of pigs [1]. When the external environmental conditions undergo drastic changes, *G. parasuis* might emerge from the upper respiratory tract, leading to infection in the host [2]. *G. parasuis* causes Glässer’s disease in pigs in the clinical setting, resulting in enormous economic losses for the pig industry [3]. The characteristics of Glässer’s disease are fibrinous polyserositis, polyarthritis and meningitis [4]. To date, 15 serovars of *G. parasuis* have been identified by the gel immunodiffusion test, but up to 20% of isolates have not been serotyped [5]. Serovars 4 and 5 have been identified as highly virulent strains [6]. In China, serotypes 4, 5 and 13 are the most prevalent [7]. Due to the serious economic losses to the breeding industry caused by high morbidity and mortality caused by *G. parasuis*, it is necessary to carry out in-depth research on how to prevent and control *G. parasuis* infection.

Currently, the inflammatory pathogenic mechanism of *G. parasuis* remains unclear. Previous research reported that the upregulation of TLR4-dependent ATP production is critical for LPS-mediated *G. parasuis* inflammation [8]. Porcine alveolar macrophage-secreted resistin inhibited the expression of the tight junction proteins claudin-5 and occludin, leading to endothelial cell dysfunction during *G. parasuis* infection [9]. *G. parasuis*-induced IL-17 expression might be positively related to pathological injury in lung and spleen tissues [10]. IL-1α, IL-1β, IL-6, IL-8, and TNF-α production and NF-κB and MAPK signalling pathway activation in porcine alveolar macrophages induced by *G. parasuis* contribute to host inflammatory responses [11]. *G. parasuis* triggered an inflammatory response in 3D4/21 cells by activating the NLRP3 inflammasome signalling pathway via ROS [12]. The sialidase of *G. parasuis* augmented the inflammatory response via desialylation and abrogation of the negative regulation of Siglec-5 [13]. *G. parasuis* breached the porcine respiratory epithelial barrier and led to lung infection by inducing autophagy and blocking the replenishment of cell membrane claudin-1 [14]. Therefore, to control inflammatory injury induced by *G. parasuis*, it is necessary to study the inflammatory pathogenic mechanism of *G. parasuis*.

Because of the excessive use of antibiotics, bacterial resistance is becoming increasingly severe and has emerged as a significant threat to public health [15]. Traditional Chinese veterinary medicine is considered a promising method for controlling inflammatory responses caused by bacteria [16]. Baicalin is isolated from the traditional medicinal plant *Scutellaria baicalensis* Georgi [17]. Baicalin has been shown to have important anti-inflammatory, antibacterial and immunomodulatory functions [18] and to attenuate cadmium-induced liver fibrosis by regulating choline metabolism [19]. Baicalin reduced lipopolysaccharide-induced intestinal inflammatory injury by suppressing the PARP1-mediated NF-κB and NLRP3 signalling pathways [20], alleviated renal fibrosis by inhibiting the PI3K/AKT/NF-κB signalling pathway [21], reduced the inflammatory-oxidative stress effect on H_2_O_2_-induced granulosa cell apoptosis via USP48 protein regulation [22] and provided protection against diethyl nitrosamine-induced liver cirrhosis by suppressing oxidative stress and inflammation [23]. Probenecid reduced infection and inflammation in acute *Pseudomonas aeruginosa* pneumonia [24]. Probenecid protected against oxygen–glucose deprivation injury in primary astrocytes by regulating inflammasome activity [25] and relieved cerebral dysfunction in sepsis by inhibiting pannexin 1-dependent ATP release [26]. Therefore, based on the biological activity of baicalin and probenecid, we investigated whether baicalin and probenecid could protect piglets induced by *G. parasuis*.

In this study, we evaluated the effects of baicalin and probenecid on protection against *G. parasuis* challenge in a piglet model. Our results showed that baicalin and probenecid protected piglets against *G. parasuis* challenge and thus might serve as a promising strategy to control and treat *G. parasuis* infection in the clinical setting.

## Materials and methods

### Ethics statement

Animal studies were approved by the Animal Care and Use Committee of Wuhan Polytechnic University, Hubei Province, China (WPU202308004). All the experimental animals were euthanized at the end of the experiment.

### Bacteria and drugs

The highly virulent serovar 5 of *G. parasuis* strain SH0165 was isolated from a commercial pig lung that displayed typical arthritis, fibrinous polyserositis, haemorrhagic pneumonia and meningitis [27]. Strain SH0165 was grown in TSB (tryptic soy broth) (Difco Laboratories, USA) or TSA (tryptic soy agar) (Difco Laboratories, USA) supplemented with 10 μg/mL NAD (Amresco, USA) and 10% foetal bovine serum (Sijiqing, Hangzhou, China) at 37 °C.

Baicalin was obtained from Sichuan Xieli Pharmaceutical Co., Ltd. (Pengzhou, Sichuan, China). Probenecid (a PANX-1 blocker) was purchased from ABclonal Technology (Wuhan, Hubei, China). ATP, ADP, AMP, UMP and UDP were purchased from Wuhan Yuanshen Bio-Tech Co., Ltd. (Wuhan, Hubei, China). N-(tert-butyldimethylsilyl)-N-methyl-trifluoroacetamide (MTBSTFA) was purchased from Shanghai Macklin Biochemical Technology Co., Ltd. (Shanghai, China).

### Experimental design

Sixty 5–6-kg, 21-day-old, naturally farrowed early-weaned (NFEW) piglets (Duroc × Landrace × large white) were purchased from Wuhan Fenglongxin Breeding Professional Cooperative (Wuhan, Hubei, China). The piglets were negative for antibodies directed against *G. parasuis* when tested with the commercial ELISA kit INGEZIM HAEMOPHILUS (INGENASA, Spain). The piglets were randomly divided into a control group, an infection group, a probenecid group and 25 mg/kg, 50 mg/kg and 100 mg/kg baicalin groups. The piglets from the probenecid group and the 25 mg/kg, 50 mg/kg and 100 mg/kg baicalin groups were injected intramuscularly with 20 mg/kg body weight (BW) probenecid and 25 mg/kg BW, 50 mg/kg BW and 100 mg/kg BW baicalin, respectively. Two hours after administration on the first day, the piglets from the infection group, the probenecid group and the 25 mg/kg, 50 mg/kg and 100 mg/kg baicalin groups were injected intraperitoneally with 1 × 10^8^ CFU of *G. parasuis* resuspended in 1 mL of TSB. The piglets in the control group were also injected intraperitoneally with an equivalent volume of TSB. After the piglets were infected with *G. parasuis* for 6 h, all the groups except the control group were injected intramuscularly with the same drugs. Subsequently, each treatment was administered twice a day for 2 days. All the piglets were monitored for 7 days, after which the survival rate was determined. The drug dosage, administration route, bacterial challenge dose, and challenge route were selected according to our previous study [28].

### Measurement of the effect of baicalin and probenecid on routine blood test indicators and changes in blood biochemical parameters

After the piglets were challenged with *G. parasuis* for 24 h, 48 h and 72 h, blood was obtained for the measurement of blood biochemical parameters and routine blood testing [29]. Blood was collected from the anterior vena cava and placed into tubes containing EDTA. Following centrifugation at 3000 × *g* for 20 min at 4 °C, the plasma was isolated from the blood for the measurement of blood biochemical parameters by utilizing commercial kits (Shanghai Kehua Bioengineering Co., Ltd., Shanghai, China). The blood biochemical parameters measured included total bilirubin (T-Bil), total protein (TP), albumin (ALB), aspartate aminotransferase (AST), alanine aminotransferase (ALT), alkaline phosphatase (ALP), total cholesterol (TC), triglycerides (TG), glucose (GLU), calcium (Ca), inorganic phosphate (IP), high-density lipoprotein cholesterol (HDL-C), low-density lipoprotein cholesterol (LDL-C), creatinine (CRE), γ-glutamyl transpeptidase (γ-GT), blood urea nitrogen (BUN) and lactate dehydrogenase (LDH). Routine blood testing was performed using anticoagulated blood. The levels of white blood cells (WBC), red blood cells (RBC), haemoglobin (HGB), platelets (PLT), neutrophils (NE), lymphocytes (LYM), monocytes (MONO) and eosinophils (EOS) were estimated by using an automatic blood analyser (Hitachi HITEC 7100, Japan).

### Detection of the effect of baicalin and probenecid on nucleoside production in piglets challenged by *G. parasuis* using LC–MS/MS

Nucleoside production in piglet blood induced by *G. parasuis* was determined by referring to our established liquid chromatography–tandem mass spectrometry (LC–MS/MS) method with some minor modifications [30]. Briefly, after the piglets were challenged with *G. parasuis* for 24 h, 48 h or 72 h, plasma was obtained and stored at − 80 °C until use. When used, the plasma was treated with 85% cold methanol (9:1, v/v), vortexed for 5 min and centrifuged (13,000 × *g*) at 4 °C for 10 min, after which the supernatant was transferred to a new 1.5 mL tube. Derivatization was initiated by placing 75 μL of MTBSTFA in 200 μL of supernatant with 85% methanol at 37 °C for 5 min with gentle vortexing. The derivatized samples were injected into the LC–MS/MS system for analysis. Nucleotide chromatography was performed by using an UltiMate 3000 UPLC system (Thermo Fisher, San Jose, CA, USA) equipped with an Acquity UPLC BEH C_18_ column (2.1 mm × 100 mm; 1.7 μm particle size; Waters, Milford, MA, USA). The mobile phase contained A (5 mM ammonium acetate aqueous solution) and B (100% acetonitrile). The column was maintained at 35 °C, and the system was operated at a flow rate of 0.15 mL/min under the following linear gradient conditions: 0–5 min, 5%–30% B; 5–10 min, 30%–95% B; 10–13 min, 95% B; 13–14 min, 95%–5% B; and 14–20 min, 5% B. MS spectra were obtained in negative ionization mode with a hybrid quadrupole orbitrap mass spectrometer (Q Exactive, Thermo Fisher). The sheath gas flow rate was 25 mL/min. The auxiliary gas flow rate was 15 mL/min. The spray voltage was 2.6 kV, and the capillary tube temperature was 320 °C. Full scan and product ion modes with an *m*/*z* range of 100–900 were employed. The quantitative data were obtained in multiple reaction monitoring (MRM) mode. Data collection was performed by using Xcalibur version 4.0 software.

### RT-PCR

After the piglets were challenged with *G. parasuis* for 48 h, blood was collected for inflammatory cytokine (IL-1β, IL-10, IL-18, TNF-α and IFN-γ) production determination. On the seventh day after the challenge, the piglets were anaesthetized and euthanized, and the aortic blood vessels were collected for protein expression measurement. The mRNA expression levels of the cytokines Panx-1, P2Y6, P2X7, NF-kB, NLRP3, Caspase-1, ROCK and MLCK were determined by RT-PCR [31]. Briefly, RNA from blood and blood vessels was isolated by using TRIzol reagent (Invitrogen, USA). Following reverse transcription into cDNA by utilizing reverse transcriptase (TaKaRa, Dalian, China), cDNA synthesis was performed by utilizing a SYBR Green PCR Kit (TaKaRa, Dalian, China) per the manufacturer’s protocol. The transcription of each sample was repeated at least three times, and β-actin was used as the internal control. The primers used in this study for RT-PCR determination are listed in Table 1.

**Table 1:** **Primer sequences for qRT-PCR analysis**

Gene		Nucleotide Sequence (5'-3')	Tm (°C)	Length (bp)
P2X7	Forward	CCCACTCTGCCATCATTGGA	57.62	88
	Reverse	GCAGACCCACCAGTAAGAGG	58.15	
P2Y6	Forward	AGCCCCTCTGGATGTAGGAG	60.11	120
	Reverse	CCGCTTGAAGTTCTCCTGGT	59.96	
Panx-1	Forward	CGCGCAGGAAATCTCAATCG	57.16	159
	Reverse	TTATGCAGGCACAGTGGGAG	57.78	
ROCK	Forward	TCCAGCTCCAGACCCTTTTG	57.48	122
	Reverse	CAGAAGGCAGTTAGCTTGGT	54.95	
MLCK	Forward	CAGCCGGTGGCAGATTGA	60.05	134
	Reverse	ATAACCGCATCACTGTCCCC	59.82	
MLC	Forward	GGCACAAATCCCACCAATGC	60.39	170
	Reverse	AAGACACGCAGACCCTCAAC	60.25	
NLRP3	Forward	CAGGCTTCTGGGACACCTTT	59.89	110
	Reverse	GTGCAGCCCTAGTCAGAGTC	59.83	
Caspase-1	Forward	TACAAGAATCCCAGGCGGTG	57.49	128
	Reverse	CCTTTGGGCTATGTCTGGGG	58.64	
NF-KB	Forward	GGCACCGGATTGAGGAGAAA	60.04	188
	Reverse	GGTGCTGAGAGATGGCGTAA	59.82	
β-actin	Forward	GAGCGCAAGTACTCCGTGT	60.08	169
	Reverse	TGCAGGTCCCGAGAGAATG	59.10	

### Western blot analysis

The protein expression levels were determined by western blotting [32]. The proteins were extracted from the piglet aortic blood vessels by utilizing a total protein extraction kit (Beyotime Biotechnology, Shanghai, China). The protein concentrations were determined by using a Bradford protein concentration assay kit according to the manufacturer’s instructions (Beyotime Biotechnology, Shanghai, China). The protein samples were separated by using 10% SDS-PAGE and transferred onto PVDF (polyvinylidene difluoride) membranes. Following blocking with 5% skim milk and washing five times with TBST, the membranes were incubated with the following primary antibodies at 4 °C overnight: anti-PANX-1 (1:1000, ABclonal Technology, Wuhan, China), anti-P2Y6 (1:2000, ABclonal), anti-P2X7 (1:1000, ABclonal), anti-P65 (1:1000, ABclonal), anti-p-P65 (1:1000, ABclonal), anti-NLRP3 (1:1000, Proteintech, Wuhan, China), anti-Caspase-1 (1:2000, Proteintech), anti-MLCK (1:1000, Absin Bioscience Inc., Shanghai, China), anti-MLC (1:1000, ABclonal), anti-p-MLC (1:1000, ABclonal), anti-ROCK (1:1000, ABclonal), anti-VE-cadherin (1:2000, ABclonal) and anti-β-actin (1:5000, Proteintech). After washing with TBST five times, the membranes were incubated with HRP-conjugated goat anti-rabbit IgG (1:10 000, Abbkine Scientific Co., Ltd., Wuhan, China) for 60 min at 37 °C, and the protein bands were visualized by using an Enhanced Chemiluminescence Detection Kit (ABclonal). The expression levels of proteins were determined using a FluorChem FC2 AIC system (Alpha Innotech, USA).

### Histopathological analysis

Blood vessels, lung, brain, spleen, and lymph nodes were collected and fixed in 10% neutral buffered formalin and hence embedded in paraffin. Then, the tissue sections were cut and stained with haematoxylin and eosin (HE) according to a standard method. The stained sections were examined using a light microscope.

### Statistical analysis

The experimental data are presented as the means ± SD. Statistical differences were determined by one-way ANOVA. Survival analysis was performed by using a log-rank test. A *p* value < 0.05 was considered significant.

## Results

### Baicalin and probenecid protect piglets against *G. parasuis* challenge

**Figure 1:**
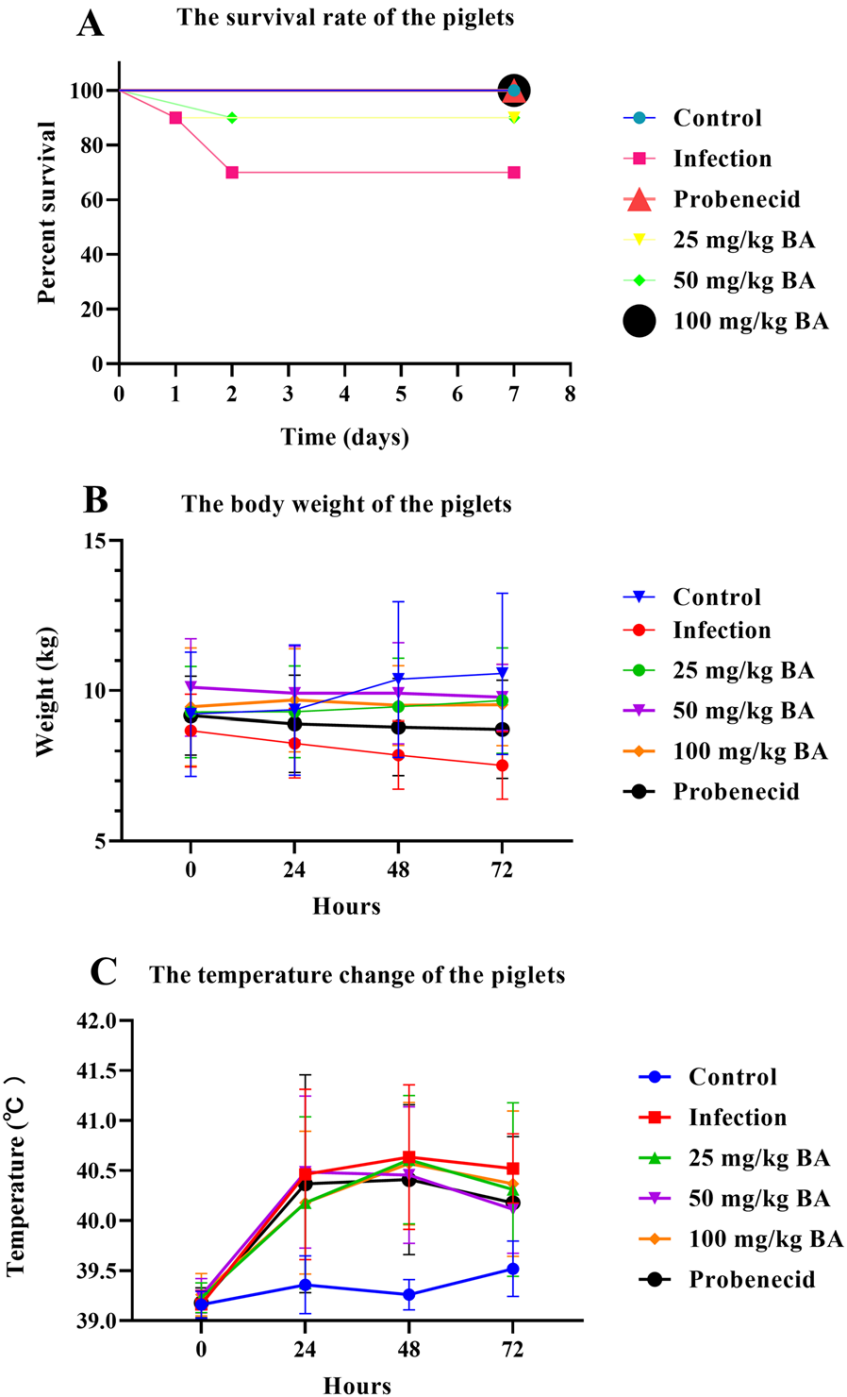
**Baicalin and probenecid provided protection against *G. parasuis*** **challenge**. After pretreatment with baicalin or probenecid, the piglets were infected with *G. parasuis*. The survival rate, body weight, and body temperature were measured. **A** Survival rate; **B** body weight change; **C** body temperature change; BA: baicalin.

After being challenged with *G. parasuis*, the piglets in the infection group had a survival rate of 70%. Probenecid, 25 mg/kg baicalin, and 50 mg/kg baicalin provided a 90% piglet survival rate against *G. parasuis* challenge, which was greater than that in the infection group (*p* < 0.05) (Figure 1A). In addition, 100 mg/kg baicalin provided 100% protection against *G. parasuis* challenge, and the survival rate was greater than that in the infection group (*p* < 0.05) (Figure 1A).

We also detected the effect of baicalin and probenecid on body weight and temperature changes after the piglets were challenged with *G. parasuis*. During the observation period, the body weights of the piglets in the infection group continuously decreased and were lower than those in the control group (*p* < 0.05) (Figure 1B). Probenecid and 25–100 mg/kg baicalin alleviated piglet weight loss compared to that in the infection group (*p* < 0.05) (Figure 1B). The temperature continuously increased in the infection group and was significantly greater than that in the control group (*p* < 0.05) (Figure 1C). Compared with infection, treatment with probenecid and 25–100 mg/kg baicalin reduced the increase in temperature (*p* < 0.05) (Figure 1C).

### Baicalin and probenecid regulate the routine blood test and blood biochemical parameters of piglets infected with *G. parasuis*

When the piglets were infected with *G. parasuis* for 24 h to 72 h, the levels of ALB, ALT, ALP, TC, TG, GLU, Ca, IP, HDL-C and LDL-C were significantly decreased, and BUN was increased in the infection group compared to the control group (Table 2, Additional files 1 and 2). Compared to the infection group, probenecid increased the levels of GLU, IP and HDL-C from 24 to 72 h (Table 2, Additional files 1 and 2). At 50–100 mg/kg, baicalin increased the levels of ALB, ALP, GLU, IP, HDL-C and LDL-C and reduced BUN compared to those in the infection group from 24 to 72 h (Table 2, Additional files 1 and 2).

**Table 2:** **Detection of the routine blood test results for 24 h**

Item	Control	GPS	20 mg/kg Probenecid	25 mg/kg BA	50 mg/kg BA	100 mg/kg BA	SEM	*p* value				
	(A)	(B)	(C)	(D)	(E)	(F)		B vs. A	C vs. B	D vs. B	E vs. B	F vs. B
WBC (10^9^/L)	26.06	15.37	20.27	24.61	26.62	32.03	1.34	< 0.001	0.009	0.001	< 0.001	< 0.001
RBC (10^9^/L)	6.59	6.62	6.23	6.03	6.75	7.07	0.14	0.816	0.492	0.682	0.907	0.600
HGB (g/L)	108.00	140.00	126.00	107.00	111.00	101.00	3.34	< 0.001	0.004	0.001	< 0.001	< 0.001
PLT (10^9^/L)	446.00	168.00	361.00	457.00	461.00	356.00	31.26	0.002	0.019	0.002	0.006	0.010
NEU (10^9^/L)	11.03	5.21	8.77	9.47	11.41	14.83	0.70	< 0.001	0.005	< 0.001	< 0.001	< 0.001
LYM (10^9^/L)	11.16	4.01	5.83	4.36	7.76	7.46	0.63	< 0.001	0.08	0.615	0.072	< 0.001
MON (10^9^/L)	2.91	2.73	3.41	2.72	2.97	5.16	0.24	0.519	0.008	0.458	0.519	< 0.001
EOS (10^9^/L)	0.28	0.39	0.41	0.36	0.40	0.42	0.02	0.001	0.735	0.109	1.000	0.109

Routine blood tests were also performed after the piglets were challenged with *G. parasuis*. At 24 h, the WBC, PLT, NE and LYM were decreased, and HGB and EOS were significantly increased compared to those in the control group, while probenecid upregulated WBC, PLT and EOS and downregulated HGB compared to those in the infection group (Additional file 3). At 50–100 mg/kg, baicalin enhanced WBC, PLT, NE and LYM and reduced HGB compared to those in the infection group (Additional file 3). From 48 to 72 h, the RBC, HGB and LYM decreased, and MONO increased in the infection group compared to the control group (Additional files 4 and 5). At 25–100 mg/kg, baicalin promoted the levels of PLT, NE and LYM and attenuated the MONO level compared to those in the infection group (Additional file 5).

### Baicalin and probenecid attenuate cytokine production in piglets infected with *G. parasuis*

**Figure 2:**
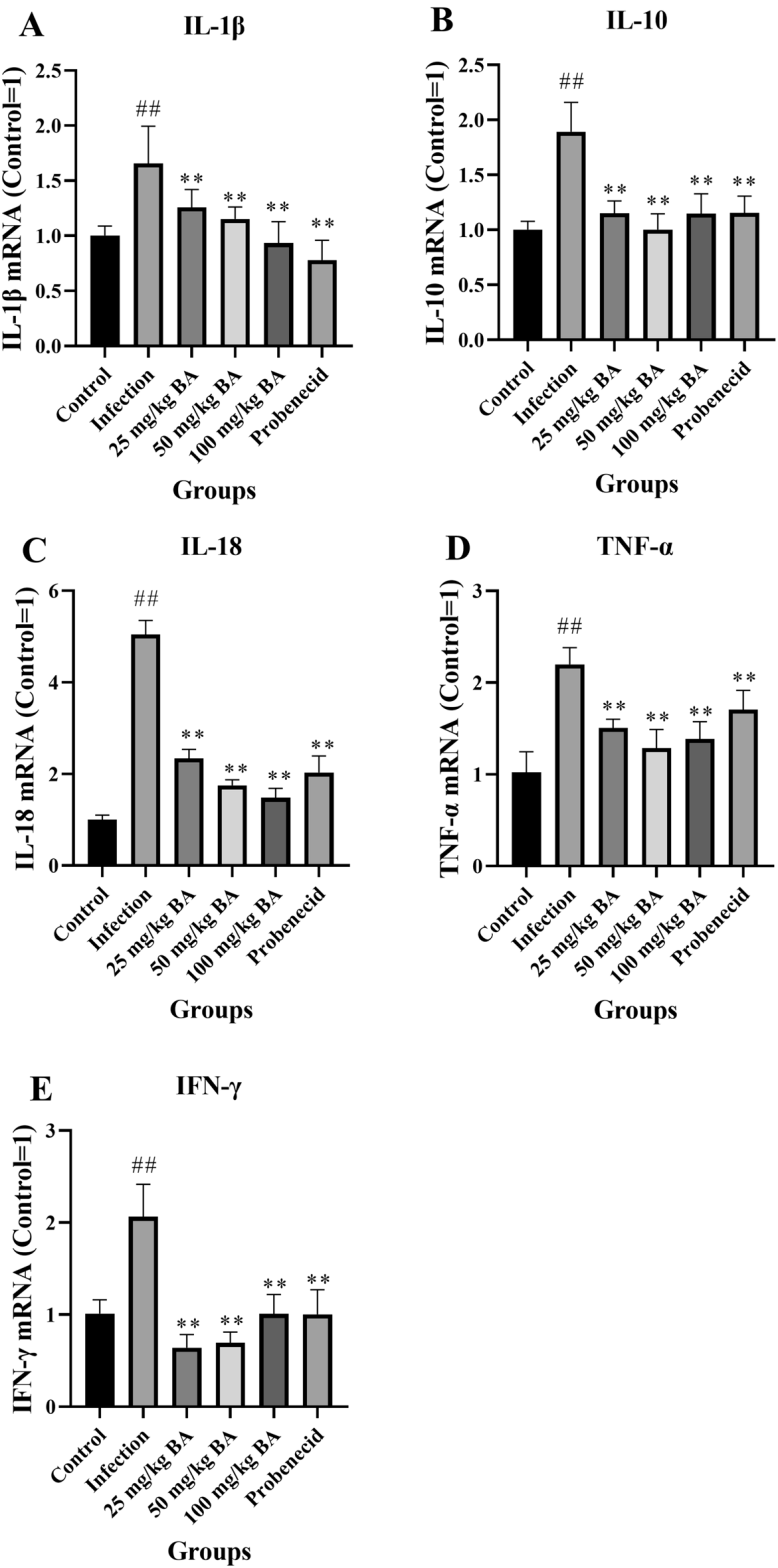
**Measurement of the effect of baicalin and probenecid on cytokine expression in *G. parasuis*** **infected piglets**. After infection for 48 h, RNA from the blood was isolated, and cytokine expression was measured by RT-PCR. **A** IL-1β; **B** IL-10; **C** IL-18; **D** TNF-α; **E** IFN-γ; BA: baicalin. ^##^*p* < 0.01 versus controls; **significance at *p* < 0.01.

After the piglets were challenged with *G. parasuis* for 48 h, the IL-1β, IL-10, IL-18, TNF-α and IFN-γ mRNA expression levels in the blood of the infection group were significantly greater than those in the blood of the control group (*p* < 0.01) (Figure 2). When the piglets were injected with baicalin and probenecid, the IL-1β, IL-10, IL-18, TNF-α and IFN-γ mRNA levels decreased compared to those in the infection group (*p* < 0.01) (Figure 2), which suggested that baicalin and probenecid inhibited cytokine production in piglets infected with *G. parasuis*.

### Baicalin and probenecid reduce Panx-1/P2Y6/P2X7 expression in the blood vessels of piglets challenged with *G. parasuis*

**Figure 3:**
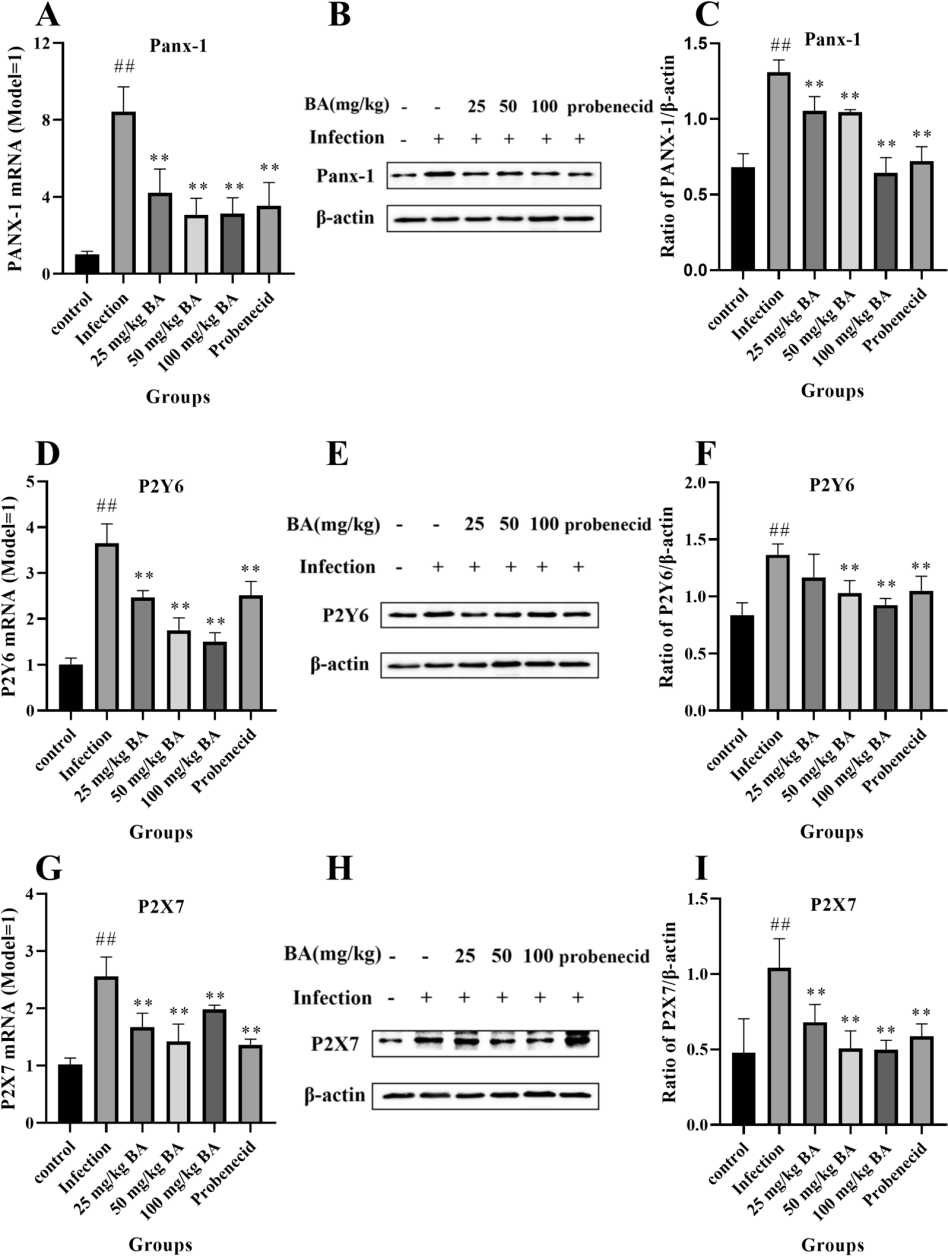
**Determination of the effect of baicalin and probenecid on Panx-1/P2Y6/P2X7 expression in the blood vessels of piglets infected with** ***G. parasuis.*** The Panx-1/P2Y6/P2X7 expression levels were determined by RT-PCR and western blotting. **A** Panx-1 mRNA level; **B, C** Panx-1 protein level; **D** P2Y6 mRNA level; **E, F** P2Y6 protein level; **G** P2X7 mRNA level; **H, I** P2X7 protein level; BA: baicalin. ^##^*p* < 0.01 versus controls; **significance at *p* < 0.01.

As shown in Figure 3, when the piglets were challenged with *G. parasuis*, the mRNA expression levels of Panx-1, P2Y6 and P2X7 were greater than those in the control group (*p* < 0.01) (Figure 3A, D, G). When the piglets were injected with probenecid or 25–100 mg/kg baicalin, the Panx-1, P2Y6 and P2X7 mRNA levels were decreased compared to those in the infection group (*p* < 0.01) (Figure 3A, D, G). *G. parasuis* also increased Panx-1, P2Y6 and P2X7 protein expression in the blood vessels compared to those in the control group (*p* < 0.01) (Figure 3B, C, E, F, H, I). Probenecid and 25–100 mg/kg baicalin decreased Panx-1, P2Y6 and P2X7 protein expression compared to that in the infection group (*p* < 0.05) (Figure 3B, C, E, F, H, I) (Panx-1, 25 mg/kg baicalin, no significant difference).

### Baicalin and probenecid inhibit the production of nucleosides in the blood of piglets challenged with *G. parasuis*

Bacterial infection can lead to the release of nucleosides [33]. The results showed that ATP, ADP, AMP and UMP were significantly upregulated in the blood of the infection group compared to the control group from 24 to 72 h (Tables 3, 4, and 5). Compared with the infection group, probenecid attenuated the production of ATP, ADP and UMP from 24 to 72 h (Tables 3, 4, and 5). At 25–100 mg/kg, baicalin weakened ATP, ADP, AMP and UMP production compared to that in the infection group (Tables 3, 4, and 5). UDP was not detected in any group from 48 to 72 h (Tables 4 and 5).

**Table 3:** **Measurement of nucleoside production in blood for 24 h**

Analyte	Control (ng/mL)	Infection (ng/mL)	25 mg/kg BA (ng/mL)	50 mg/kg BA (ng/mL)	100 mg/kg BA (ng/mL)	Probenecid (ng/mL)
ATP	ND	827.26	ND	ND	ND	ND
ADP	4.87	56.39^##^	5.45**	4.27**	2.76**	3.40**
AMP	584.87	1281.99^##^	415.12**	503.76**	398.84**	474.19**
UMP	ND	11.77	ND	ND	2.00*	ND
UDP	ND	34.26	ND	ND	ND	ND

#*p*<0.05 versus controls; ##*p*<0.01 versus controls; *significance at *p*<0.05; **significance at *p*<0.01

ND: not detected.

**Table 4:** **Measurement of nucleoside production in blood for 48 h**

Analyte	Control (ng/mL)	Infection (ng/mL)	25 mg/kg BA (ng/mL)	50 mg/kg BA (ng/mL)	100 mg/kg BA (ng/mL)	Probenecid (ng/mL)
ATP	ND	1394.24	ND	327.67*	335.72*	ND
ADP	ND	39.36	7.72**	9.15**	ND	6.31**
AMP	571.24	1350.54^##^	765.83**	929.37**	652.46**	590.09**
UMP	0.58	6.21^#^	1.37	3.07*	ND	2.36*
UDP	ND	ND	ND	ND	ND	ND

#*p*<0.05 versus controls; ##*p*<0.01 versus controls; *significance at *p*<0.05; **significance at *p*<0.01

ND: not detected.

**Table 5:** **Measurement of nucleoside production in blood for 72 h**

Analyte	Control (ng/mL)	Infection (ng/mL)	25 mg/kg BA (ng/mL)	50 mg/kg BA (ng/mL)	100 mg/kg BA (ng/mL)	Probenecid (ng/mL)
ATP	ND	182.92	ND	36.37	57.81	61.45
ADP	0.28	47.15^##^	24.66**	34.72**	14.08**	34.35**
AMP	497.14	845.51^#^	776.97	564.71*	604.37*	787.34
UMP	ND	30.79	7.10**	6.44**	4.85**	5.55**
UDP	ND	ND	ND	ND	ND	ND

#*p*<0.05 versus controls; ##*p*<0.01 versus controls; *significance at *p*<0.05; **significance at *p*<0.01

ND not detected.

### Baicalin and probenecid weaken NF-kB, AP-1 and NLRP3/Caspase-1 signalling activation in blood vessels of piglets infected with *G. parasuis*

**Figure 4:**
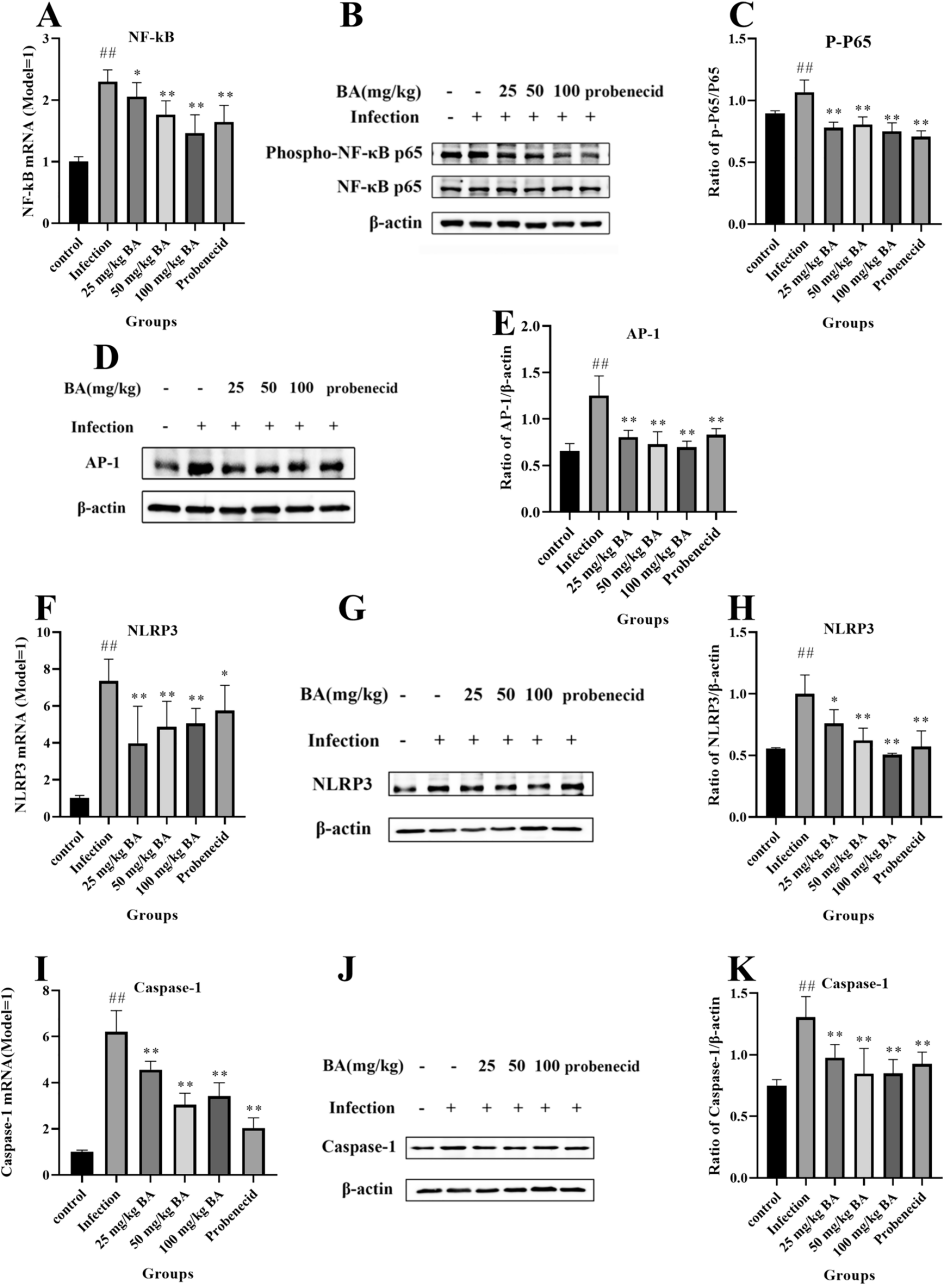
**Measurement of the effect of baicalin and probenecid on NF-κB, AP-1 and NLRP3/Caspase-1 signalling activation in blood vessels of piglets infected with *G. parasuis.*** The expression levels of NF-kB, AP-1, NLRP3, and Caspase-1 were determined by RT-PCR and western blotting. **A** NF-κB mRNA; **B, C** P-P65 protein; **D, E** AP-1 protein; **F** NLRP3 mRNA; **G, H** NLRP3 protein; **I**: Caspase-1 mRNA; **J, K** Caspase-1 protein; BA: Baicalin. ^##^*p* < 0.01 versus controls; *significance at *p* < 0.05; **significance at *p* < 0.01.

NF-kB, AP-1 and NLRP3/Caspase-1 signalling are involved in tissue inflammation and injury during disease processes [34]. As shown in Figure 4A, F, I, G*. parasuis* upregulated NF-kB, NLRP3 and Caspase-1 mRNA expression compared to that in the control group (*p* < 0.01). Probenecid and 25–100 mg/kg baicalin inhibited the mRNA levels of NF-kB, NLRP3 and Caspase-1 compared to those in the infection group (*p* < 0.05) (Figure 4A, F, I). The NF-kB, AP-1, NLRP3 and Caspase-1 expression levels were measured by western blotting. The p-p65, AP-1, NLRP3 and Caspase-1 protein expression levels were upregulated in the control group compared to those in the control group, while the p-p65, AP-1, NLRP3 and Caspase-1 protein expression levels were decreased in the probenecid group and the baicalin groups compared to those in the infection group (*p* < 0.05) (Figure 4B, C, D, E, G, H, J, K).

### Baicalin and probenecid attenuate ROCK/MLCK/MLC activation in the blood vessels of piglets infected with *G. parasuis*

**Figure 5:**
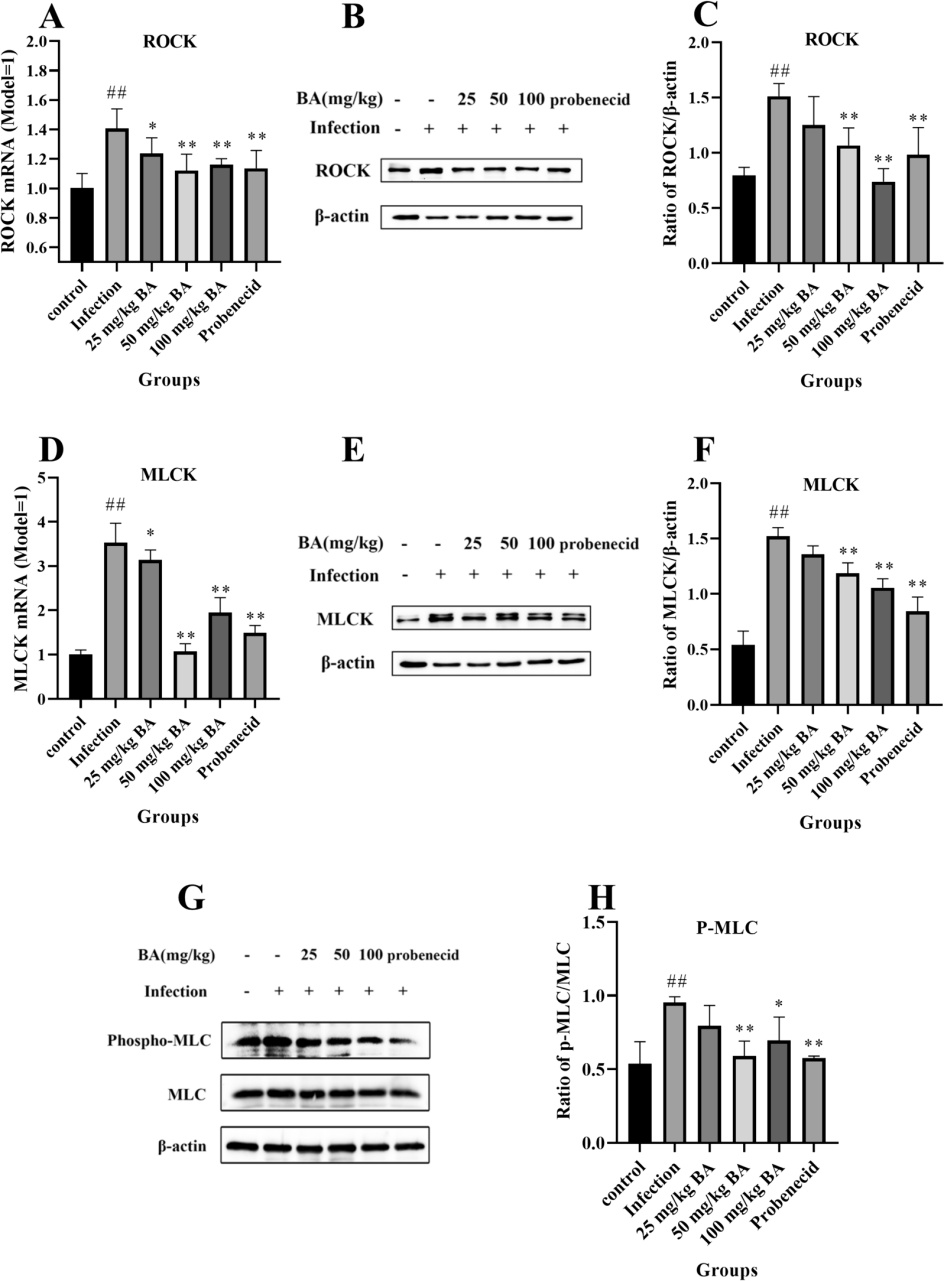
**Measurement of the effect of baicalin and probenecid on ROCK/MLCK/MLC activation in the blood vessels of piglets infected with *G. parasuis.*** The ROCK/MLCK/MLC expression levels were explored by RT-PCR and western blotting. **A** ROCK mRNA level; **B, C** ROCK protein level; **D** MLCK mRNA level; **E, F** MLCK protein level; **G, H** P-MLC protein level; BA: baicalin. ^##^*p* < 0.01 versus controls; *significance at *p* < 0.05; **significance at *p* < 0.01.

The results demonstrated that the mRNA expression levels of ROCK and MLCK in the infection group were significantly greater than those in the control group (*p* < 0.01) (Figure 5A and D). However, compared with the infection group, treatment with probenecid or 25–100 mg/kg baicalin decreased ROCK and MLCK mRNA expression (*p* < 0.05) (Figure 5A and D). We also determined ROCK, MLCK and MLC protein expression levels in blood vessels after *G. parasuis* challenge. As shown in Figure 5B, C, E, F, G, and H, compared to those in the control group, ROCK, MLCK and p-MLC/MLC were upregulated in the infection group (*p* < 0.01). Compared with infection alone, probenecid inhibited ROCK, MLCK and MLC protein expression (*p* < 0.01) (Figure 5B, C, E, F, G, H). Furthermore, 25–100 mg/kg baicalin attenuated the protein expression levels of ROCK and MLCK (*p* < 0.01) (Figure 5B, C, E, F, G, H), and 50–100 mg/kg baicalin reduced the protein expression level of MLC compared to that in the infection group (*p* < 0.05) (Figure 5B, C, E, F, G, H).

### Baicalin and probenecid upregulated VE-cadherin expression in the blood vessels of piglets challenged with *G. parasuis*

**Figure 6:**
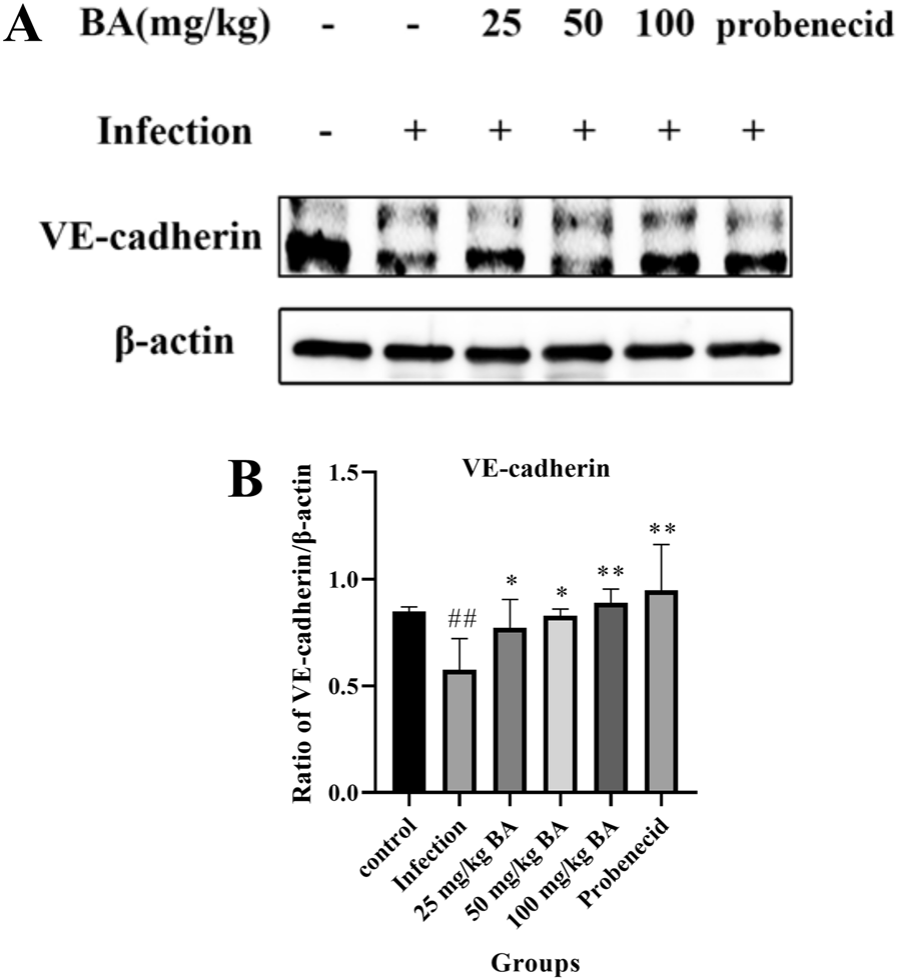
**The effect of baicalin and probenecid on VE-cadherin expression in the blood vessels of piglets challenged with *G. parasuis.*** The VE-cadherin expression level was explored by western blot. **A, B** VE-cadherin protein level; BA: baicalin. ^##^*p* < 0.01 versus controls; *significance at *p* < 0.05; **significance at *p* < 0.01.

VE-cadherin plays a key role in governing the endothelial barrier [35]; therefore, we investigated the effect of baicalin and probenecid on VE-cadherin regulation in blood vessels. The protein expression level of VE-cadherin was significantly lower than that in the control group (*p* < 0.01) (Figure 6A and B). Compared with infection alone, probenecid promoted VE-cadherin protein expression (*p* < 0.01) (Figure 6A and B). In addition, 25–100 mg/kg baicalin enhanced VE-cadherin protein expression compared to that in the infection group (*p* < 0.05) (Figure 6A and B).

### Baicalin and probenecid alleviate pathological tissue damage in piglets infected with *G. parasuis*

**Figure 7:**
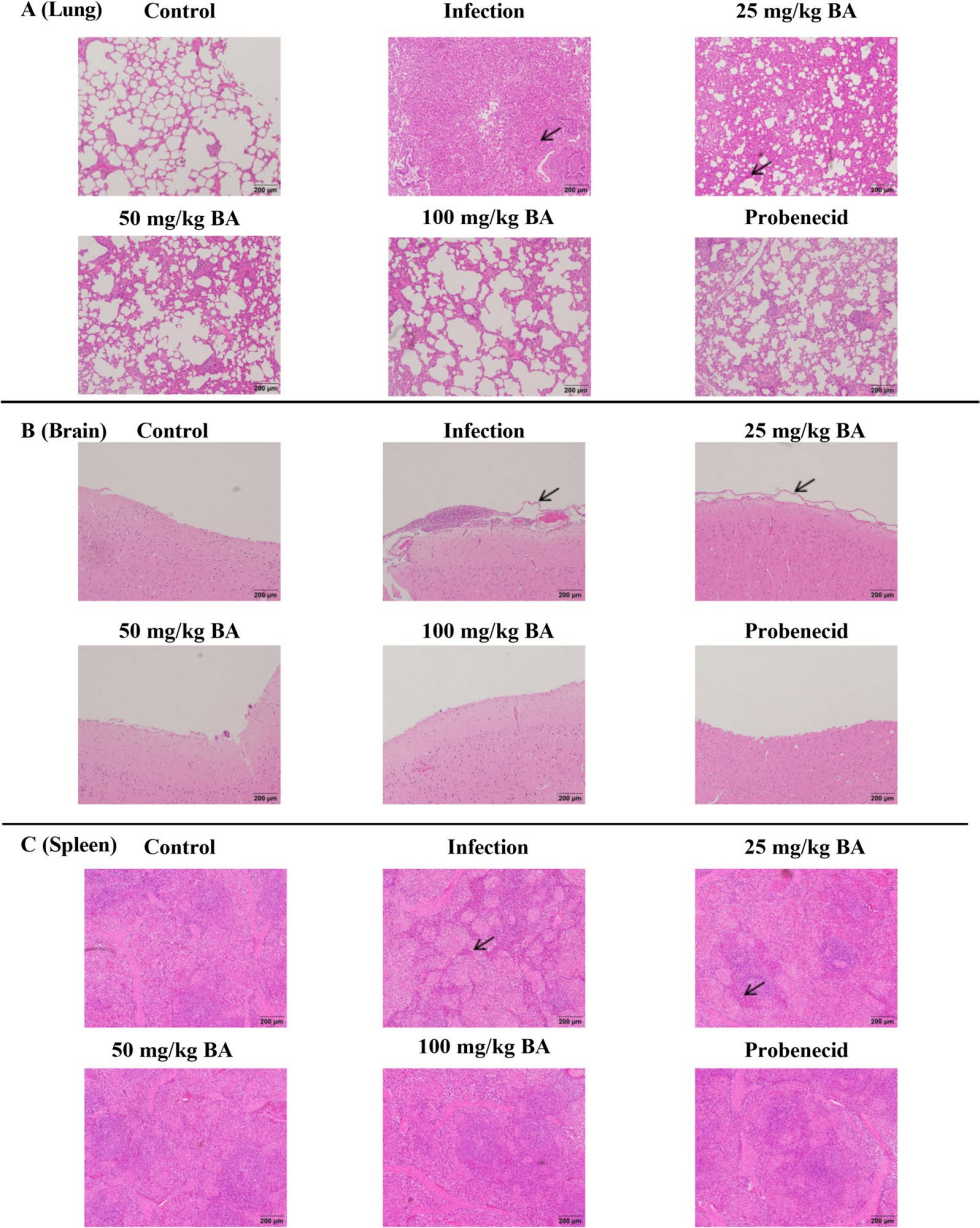
**The effect of baicalin and probenecid on alleviating pathological tissue damage in piglets infected with *G. parasuis.*** The lung, brain, and spleen were harvested to prepare tissue sections. A: lung; B: brain; C: spleen; BA: baicalin.

As shown in Figure 7 and Additional file 6, there was severe pathological tissue damage in the piglets from the infection group compared to those from the control group. The characteristics of lung histological damage in the infection group were oedema, haemorrhage and inflammatory cell infiltration (Figure 7A). The infection group showed meningeal detachment, cerebral haemorrhage and inflammatory cell infiltration in the brain (Figure 7B), as well as red pulp hyperaemia and white pulp haemorrhage in the spleen (Figure 7C). However, only minor damage was present in the probenecid and baicalin groups (Figure 7). Similar results were observed for the blood vessels and lymph nodes (Additional file 6). These results suggested that baicalin and probenecid reduced *G. parasuis*-induced pathological tissue damage in piglets.

## Discussion

*G. parasuis* is a commensal bacterium that colonizes the upper respiratory tract of healthy pigs and can cause Glässer’s disease under stress conditions [36]. Infection of piglets with *G. parasuis* induced systemic inflammation [37]. However, the mechanism of vascular inflammation remains unclear. In this study, we investigated whether baicalin and probenecid protect against *G. parasuis* challenge in a piglet model. Our results might provide a new method for controlling *G. parasuis* infection.

In this study, we used intramuscular injection as the route of administration. In our previous study, we determined the pharmacokinetics of baicalin in plasma by intravenous and intramuscular administration of baicalin to piglets and found that intramuscular injection of baicalin in piglets elicited more rapid and complete absorption than intravenous injection [38]. Therefore, intramuscular injection is an efficient administration route for treating infectious diseases in piglets. Pannexin 1 (PANX-1), a large-pore membrane channel, is highly permeable to ATP [39]. PANX-1 has been shown to play key roles in the regulation of leukocyte trafficking and tissue inflammation [40]. Previous research has shown the expression of PANX-1 in blood vessels [41]. However, the functions of PANX-1 in blood vessels are not clear. Pannexin-1 channels on endothelial cells modulate ATP secretion to regulate the pathogenesis of abdominal aortic aneurysm formation [42]. PANX-1 mediates necrosis-induced NLRP3 inflammasome activation in macrophages during acute kidney injury [43]. PANX-1 is considered a driver of inflammation and ischaemia‒reperfusion injury [44]. Our previous study demonstrated that *G. parasuis* upregulated the expression of the PANX-1 channel protein, promoted the release of nucleosides in porcine aortic vascular endothelial cells (PAVEC) and activated the MLCK/MLC pathway, leading to PAVEC damage [30]. However, whether *G. parasuis* induces PANX-1 expression and nucleoside release in piglets has not been investigated. Our results revealed that *G. parasuis* triggered PANX-1 expression in blood vessels and nucleoside production in the blood, activated the ROCK/MLCK/MLC pathway and downregulated VE-cadherin expression in blood vessels, which might be an important mechanism by which *G. parasuis* causes vascular inflammation. Probenecid, a PANX-1 blocker, inhibited the release of intracellular ATP to the extracellular compartment during inflammation [45]. Probenecid attenuated renal injury through synergistic inhibition of the PANX-1/P2X7R axis [46]. Blocking the function of PANX-1 pharmacologically (with baicalin and probenecid) greatly reduced *G. parasuis*-induced nucleoside release. Our study is the first to detect nucleoside substances released into the blood of piglets infected with *G. parasuis*, providing new drug targets for controlling *G. parasuis* infection.

The NF-κB/AP-1/NLRP3 pathways play important roles in bacterium-induced inflammatory responses. *Escherichia coli* K88 impaired intestinal integrity and induced inflammation via the NF-κB pathway [47]. Costunolide attenuated LPS-induced inflammation and lung injury by inhibiting NF-κB signalling [48]. Limonene exerted an anti-inflammatory effect on LPS-induced jejunal injury in mice by inhibiting the NF-κB/AP-1 pathway [49]. Liciritin, which targets Th17 cell differentiation and abnormal proliferation of keratinocytes, alleviated psoriasis via the NF-κB and AP-1 pathways [50]. Polyethylene microplastics induce gut microbiota dysbiosis, leading to liver injury via the NF-κB/NLRP3 pathway in mice [51]. Naringin alleviated the inflammatory response caused by *Actinobacillus pleuropneumoniae* by downregulating the NF-κB/NLRP3 signalling pathway [52]. Fusidic acid derivatives alleviate acute lung injury by inhibiting the NF-κB/NLRP3 pathway [53]. BtpB inhibited innate inflammatory responses in goat alveolar macrophages through the NF-κB and NLRP3 pathways during *Brucella* infection [54]. In this study, baicalin and probenecid alleviated *G. parasuis*-induced tissue injury and inflammation by inhibiting NF-κB/AP-1/NLRP3 activation and inflammatory cytokine expression, which makes them potential candidates for the treatment of inflammatory diseases.

In this study, our results demonstrated that baicalin and probenecid inhibited ROCK/MLCK/MLC pathway activation in the blood vessels of *G. parasuis*-challenged piglets. Other studies reported that naringin attenuated sepsis-induced intestinal injury via the ROCK/NF-κB/MLCK/MLC signalling pathway [55]. Ruscogenin ameliorated dasatinib-induced intestinal barrier dysfunction in HUVECs via the ROCK/MLC pathway [56]. QiShenYiQi pills reduced ischaemia/reperfusion-induced cardiac microvascular hyperpermeability, implicating ROCK/MLC signalling [57]. Perlecan improved spinal cord barrier repair through the ROCK/MLC pathway after spinal cord injury [58]. Palmitoylethanolamide prevented early BBB disruption after cerebral ischaemic/reperfusion injury through the regulation of ROCK/MLC signalling [59]. Because numerous traditional Chinese veterinary drugs can improve inflammatory responses by inhibiting the ROCK/MLCK/MLC signalling pathway, targeting the ROCK/MLCK/MLC pathway might be a viable option for ameliorating *G. parasuis*-induced inflammation.

Taken together, our results demonstrate that baicalin and probenecid provide greater protection against *G. parasuis* challenge in piglets. Baicalin and probenecid inhibited IL-1β, IL-10, IL-18, TNF-α and IFN-γ expression, attenuated Panx-1/P2Y6/P2X7 expression, reduced NF-kB/AP-1/NLRP3/Caspase-1 and ROCK/MLCK/MLC pathway activation, enhanced VE-cadherin expression in *G. parasuis*-induced blood vessels of piglets and improved pathological tissue damage in piglets triggered by *G. parasuis*. Our findings reveal novel targets for controlling *G. parasuis* infection.

### Supplementary Information

**Additional file 1. Routine blood tests were performed for 48 h.**

**Additional file 2. Routine blood tests were performed for 72 h.**

**Additional file 3. Blood biochemical parameters were detected for 24 h.**

**Additional file 4. Blood biochemical parameters were detected for 48 h.**

**Additional file 5. Blood biochemical parameters were detected for 72 h.**

**Additional file 6. The effect of baicalin and probenecid on alleviating pathological tissue damage in piglets infected with *G. parasuis.*** Blood vessels and lymph nodes were obtained to prepare tissue sections. A: Blood vessels; B: lymph node; BA: baicalin.

## Data Availability

The data supporting the conclusions of this article are included within the article. Additional data used and/or analysed during the current study are available from the corresponding author upon reasonable request.
