# Supplementary material for: Baicalin and probenecid protect against Glaesserella parasuis challenge in a piglet model
Source: Vet Res. 2024 Jul 29;55:96. doi: 10.1186/s13567-024-01352-4 (PMC11285411; doi:10.1186/s13567-024-01352-4)
Supplement: Supplementary file 1 — Additional file 1. Routine blood tests were performed for 48 h. [file 13567_2024_1352_MOESM1_ESM.docx]

**Additional file 1** **Detection of the routine blood test for 48 h**

| Item | Control | GPS | 20 mg/kg  Probenecid | 25 mg/kg BA | 50 mg/kg BA | 100 mg/kg BA | SEM | *P* value | | | | |
| --- | --- | --- | --- | --- | --- | --- | --- | --- | --- | --- | --- | --- |
|  | (A) | (B) | (C) | (D) | (E) | (F) |  | Bvs.A | C vs. B | D vs. B | E vs. B | F vs. B |
| WBC (109/L) | 22.19 | 14.05 | 25.02 | 21.08 | 22.12 | 23.50 | 1.01 | <0.001 | <0.001 | <0.001 | <0.001 | <0.001 |
| RBC (109/L) | 6.88 | 4.76 | 5.17 | 5.88 | 6.43 | 5.38 | 0.17 | 0.001 | 0.205 | 0.207 | 0.013 | 0.151 |
| HGB (g/L) | 93.00 | 89.00 | 92.00 | 105.00 | 111.00 | 118.00 | 2.07 | 0.912 | 0.825 | 0.219 | 0.128 | 0.026 |
| PLT (109/L) | 507.00 | 222.00 | 503.00 | 390.00 | 367.00 | 370.00 | 34.11 | 0.005 | 0.003 | 0.015 | 0.206 | 0.012 |
| NEU (109/L) | 8.1 | 6.57 | 15.30 | 8.47 | 5.89 | 12.11 | 0.85 | 0.205 | 0.001 | 0.192 | 0.863 | 0.040 |
| LYM (109/L) | 9.12 | 5.45 | 9.89 | 9.64 | 10.72 | 9.94 | 0.51 | 0.033 | 0.041 | 0.133 | 0.007 | 0.051 |
| MON (109/L) | 1.74 | 7.01 | 3.77 | 4.5 | 5.50 | 2.39 | 0.65 | 0.001 | 0.014 | 0.003 | 0.034 | 0.001 |
| EOS (109/L) | 0.27 | 0.60 | 0.51 | 0.57 | 0.52 | 0.53 | 0.04 | 0.002 | 0.263 | 0.24 | 0.219 | 0.209 |
