# Supplementary material for: Baicalin and probenecid protect against Glaesserella parasuis challenge in a piglet model
Source: Vet Res. 2024 Jul 29;55:96. doi: 10.1186/s13567-024-01352-4 (PMC11285411; doi:10.1186/s13567-024-01352-4)
Supplement: Supplementary file 2 — Additional file 2. Routine blood tests were performed for 72 h. [file 13567_2024_1352_MOESM2_ESM.docx]

**Additional file 2** **Detection of the routine blood test for 72 h**

| Item | Control | GPS | 20 mg/kg  Probenecid | 25 mg/kg BA | 50 mg/kg BA | 100 mg/kg BA | SEM | *P* value | | | | |
| --- | --- | --- | --- | --- | --- | --- | --- | --- | --- | --- | --- | --- |
|  | (A) | (B) | (C) | (D) | (E) | (F) |  | Bvs. A | C vs. B | D vs. B | E vs. B | F vs. B |
| WBC (109/L) | 21.61 | 19.92 | 24.58 | 21.06 | 20.36 | 21.33 | 0.76 | 0.938 | 0.054 | 0.671 | 0.804 | 0.163 |
| RBC (109/L) | 5.97 | 5.10 | 5.40 | 5.36 | 6.05 | 7.49 | 0.21 | 0.069 | 0.918 | 0.67 | 0.1 | <0.001 |
| HGB (g/L) | 86.00 | 92.00 | 97.00 | 94.00 | 99.00 | 93.00 | 1.44 | 0.814 | 0.725 | 0.814 | 0.417 | 0.725 |
| PLT (109/L) | 438.00 | 186.00 | 356.00 | 393.00 | 455.00 | 329.00 | 25.97 | <0.001 | 0.008 | 0.006 | <0.001 | 0.016 |
| NEU (109/L) | 9.41 | 4.80 | 12.41 | 7.78 | 8.32 | 10.91 | 0.94 | 0.001 | <0.001 | 0.008 | 0.026 | <0.001 |
| LYM (109/L) | 10.32 | 4.07 | 7.00 | 8.74 | 9.26 | 7.11 | 0.55 | 0.002 | 0.064 | 0.014 | 0.004 | 0.033 |
| MON (109/L) | 3.03 | 12.03 | 8.54 | 4.28 | 8.29 | 9.01 | 0.85 | <0.001 | 0.017 | 0.001 | 0.026 | 0.013 |
| EOS (109/L) | 0.33 | 0.46 | 0.28 | 0.42 | 0.51 | 0.55 | 0.34 | 0.214 | 0.014 | 0.748 | 0.145 | 0.451 |
