# Supplementary material for: Baicalin and probenecid protect against Glaesserella parasuis challenge in a piglet model
Source: Vet Res. 2024 Jul 29;55:96. doi: 10.1186/s13567-024-01352-4 (PMC11285411; doi:10.1186/s13567-024-01352-4)
Supplement: Supplementary file 3 — Additional file 3. Blood biochemical parameters were detected for 24 h. [file 13567_2024_1352_MOESM3_ESM.docx]

| Item | Control | GPS | 25 mg/kg BA | 50 mg/kg BA | 100 mg/kg BA | 20 mg/kg Probenecid | SEM | P value | | | | |
| --- | --- | --- | --- | --- | --- | --- | --- | --- | --- | --- | --- | --- |
|  | (A) | (B) | (C) | (D) | (E) | (F) |  | B vs. A | C vs. B | D vs. B | E vs. B | F vs. B |
| T-Bil (μmol/L) | 0.47 | 1.63 | 1.24 | 0.85 | 0.72 | 1.20 | 0.16 | 0.052 | 0.480 | 0.174 | 0.116 | 0.441 |
| TP (g/L) | 46.36 | 43.92 | 44.11 | 46.01 | 45.32 | 46.05 | 0.30 | 0.009 | 0.819 | 0.021 | 0.100 | 0.019 |
| ALB (g/L) | 23.52 | 17.97 | 19.30 | 21.59 | 22.02 | 20.31 | 0.48 | <0.001 | 0.083 | <0.001 | <0.001 | 0.006 |
| AST (U/L) | 99.00 | 83.00 | 85.00 | 91.00 | 94.00 | 93.67 | 3.35 | 0.241 | 0.918 | 0.558 | 0.432 | 0.432 |
| ALT (U/L) | 77.00 | 51.00 | 62.00 | 67.00 | 65.00 | 56.00 | 3.08 | 0.019 | 0.261 | 0.115 | 0.162 | 0.612 |
| ALP (U/L) | 466.00 | 266.00 | 370.00 | 398.00 | 424.00 | 417.00 | 16.31 | <0.001 | 0.001 | <0.001 | <0.001 | <0.001 |
| TC (mmol/L) | 2.00 | 1.19 | 1.52 | 1.80 | 1.96 | 1.61 | 0.08 | 0.002 | 0.134 | 0.013 | 0.003 | 0.066 |
| TG (mmol/L) | 0.55 | 0.38 | 0.41 | 0.53 | 0.56 | 0.51 | 0.02 | 0.038 | 0.689 | 0.068 | 0.033 | 0.109 |
| GLU (mmol/L) | 5.40 | 1.57 | 3.33 | 3.67 | 4.03 | 3.67 | 0.31 | <0.001 | 0.017 | 0.006 | 0.002 | 0.006 |
| Ca (mmol/L) | 2.51 | 2.23 | 2.39 | 2.44 | 2.54 | 2.38 | 0.03 | 0.015 | 0.138 | 0.055 | 0.009 | 0.145 |
| IP (mmol/L) | 3.13 | 2.45 | 3.00 | 3.05 | 3.08 | 3.03 | 0.07 | 0.002 | 0.009 | 0.005 | 0.003 | 0.006 |
| CRE (μmol/L) | 0.94 | 0.19 | 0.77 | 0.93 | 0.87 | 0.55 | 0;07 | <0.001 | <0.001 | <0.001 | <0.001 | 0.003 |
| HDL-C (mmol/L) | 1.14 | 0.70 | 0.95 | 1.04 | 1.03 | 0.84 | 0.04 | <0.001 | 0.014 | 0.002 | 0.002 | 0.124 |
| LDL-C (mmol/L) | 78.27 | 78.79 | 76.21 | 75.76 | 78.65 | 77.73 | 2.12 | 0.953 | 0.770 | 0.731 | 0.987 | 0.904 |
| UA (μmol/L) | 41.00 | 91.00 | 66.00 | 49.00 | 47.00 | 43.67 | 4.32 | <0.001 | <0.001 | <0.001 | <0.001 | <0.001 |
| γ-GT (U/L) | 4.10 | 7.97 | 5.07 | 4.43 | 4.33 | 5.33 | 0.44 | 0.008 | 0.036 | 0.014 | 0.012 | 0.053 |
| CK (U/L) | 1045.00 | 1169.00 | 1106.00 | 1131.00 | 1074.00 | 1029.00 | 23.90 | 0.172 | 0.474 | 0.665 | 0.290 | 0.126 |

**Additional file 3** **Detection of the blood biochemical parameters for 24 h**
