# Supplementary material for: Baicalin and probenecid protect against Glaesserella parasuis challenge in a piglet model
Source: Vet Res. 2024 Jul 29;55:96. doi: 10.1186/s13567-024-01352-4 (PMC11285411; doi:10.1186/s13567-024-01352-4)
Supplement: Supplementary file 4 — Additional file 4. Blood biochemical parameters were detected for 48 h. [file 13567_2024_1352_MOESM4_ESM.docx]

**Additional file 4** **Detection of the blood biochemical parameters for 48 h**

| Item | Control | GPS | 25 mg/kg BA | 50 mg/kg BA | 100 mg/kg BA | 20 mg/kg Probenecid | SEM | P value | | | | |
| --- | --- | --- | --- | --- | --- | --- | --- | --- | --- | --- | --- | --- |
|  | (A) | (B) | (C) | (D) | (E) | (F) |  | B vs. A | C vs. B | D vs. B | E vs. B | F vs. B |
| T-Bil (μmol/L) | 0.45 | 3.73 | 0.44 | 2.42 | 2.90 | 2.64 | 0.30 | <0.001 | <0.001 | <0.001 | <0.001 | <0.001 |
| TP (g/L) | 47.53 | 44.31 | 46.08 | 46.60 | 45.18 | 47.88 | 0.35 | 0.001 | 0.030 | 0.008 | 0.248 | <0.001 |
| ALB (g/L) | 23.62 | 18.25 | 21.59 | 20.44 | 21.11 | 19.73 | 0.40 | <0.001 | <0.001 | <0.001 | <0.001 | <0.001 |
| AST (U/L) | 100.67 | 73.00 | 72.67 | 81.33 | 90.67 | 110.33 | 3.50 | <0.001 | 0.926 | 0.036 | <0.001 | <0.001 |
| ALT (U/L) | 71.33 | 45.67 | 62.00 | 49.67 | 64.33 | 69.00 | 2.37 | <0.001 | <0.001 | 0.093 | <0.001 | <0.001 |
| ALP (U/L) | 470.33 | 186.67 | 169.33 | 159.00 | 210.67 | 194.67 | 26.46 | <0.001 | 0.287 | 0.100 | 0.149 | 0.616 |
| TC (mmol/L) | 1.95 | 1.10 | 1.31 | 1.54 | 1.03 | 1.60 | 0.08 | <0.001 | 0.057 | 0.001 | 0.524 | <0.001 |
| TG (mmol/L) | 0.56 | 0.60 | 0.69 | 0.59 | 0.54 | 0.82 | 0.03 | 0.444 | 0.112 | 0.846 | 0.211 | 0.001 |
| GLU (mmol/L) | 5.63 | 1.90 | 2.80 | 4.17 | 4.10 | 4.93 | 0.31 | <0.001 | 0.014 | <0.001 | <0.001 | <0.001 |
| Ca (mmol/L) | 2.53 | 2.26 | 2.37 | 2.55 | 2.46 | 2.39 | 0.03 | 0.003 | 0.186 | 0.002 | 0.019 | 0.120 |
| IP (mmol/L) | 3.29 | 2.21 | 2.61 | 2.42 | 2.90 | 2.94 | 0.09 | <0.001 | 0.001 | 0.047 | <0.001 | <0.001 |
| CRE (μmol/L) | 0.95 | 0.17 | 0.51 | 0.63 | 0.73 | 0.72 | 0.06 | <0.001 | <0.001 | <0.001 | <0.001 | <0.001 |
| HDL-C (mmol/L) | 1.19 | 0.56 | 0.87 | 0.71 | 0.77 | 0.83 | 0.05 | <0.001 | <0.001 | <0.001 | <0.001 | <0.001 |
| LDL-C (mmol/L) | 77.66 | 73.58 | 72.58 | 77.32 | 65.43 | 69.24 | 1.44 | 0.334 | 0.809 | 0.375 | 0.067 | 0.305 |
| UA (μmol/L) | 41.67 | 43.00 | 46.00 | 45.67 | 44.00 | 46.67 | 0.78 | 0.631 | 0.290 | 0.344 | 0.718 | 0.201 |
| γ-GT (U/L) | 4.53 | 8.33 | 3.57 | 4.87 | 3.50 | 3.60 | 0.43 | <0.001 | <0.001 | <0.001 | <0.001 | <0.001 |
| CK (U/L) | 1103.90 | 949.53 | 895.47 | 872.80 | 885.23 | 849.03 | 21.41 | <0.001 | 0.038 | 0.006 | 0.017 | 0.001 |
