# Supplementary material for: Baicalin and probenecid protect against Glaesserella parasuis challenge in a piglet model
Source: Vet Res. 2024 Jul 29;55:96. doi: 10.1186/s13567-024-01352-4 (PMC11285411; doi:10.1186/s13567-024-01352-4)
Supplement: Supplementary file 5 — Additional file 5. Blood biochemical parameters were detected for 72 h. [file 13567_2024_1352_MOESM5_ESM.docx]

**Additional file 5** **Detection of the blood biochemical parameters for 72 h**

| Item | Control | GPS | 25 mg/kg BA | 50 mg/kg BA | 100 mg/kg BA | 20 mg/kg Probenecid | SEM | P value | | | | |
| --- | --- | --- | --- | --- | --- | --- | --- | --- | --- | --- | --- | --- |
|  | (A) | (B) | (C) | (D) | (E) | (F) |  | B vs. A | C vs. B | D vs. B | E vs. B | F vs. B |
| T-Bil (μmol/L) | 0.46 | 1.61 | 0.91 | 0.67 | 0.64 | 0.72 | 0.13 | 0.009 | 0.078 | 0.024 | 0.021 | 0.032 |
| TP (g/L) | 45.92 | 48.13 | 46.66 | 48.93 | 47.29 | 48.08 | 0.30 | 0.009 | 0.059 | 0.282 | 0.254 | 0.941 |
| ALB (g/L) | 22.78 | 15.08 | 19.01 | 19.64 | 19.87 | 18.51 | 0.61 | <0.001 | 0.003 | 0.001 | 0.001 | 0.007 |
| AST (U/L) | 106.00 | 77.00 | 86.00 | 84.00 | 104.00 | 78.00 | 3.68 | 0.009 | 0.329 | 0.455 | 0.013 | 0.918 |
| ALT (U/L) | 75.00 | 37.00 | 66.00 | 69.00 | 63.00 | 60.00 | 3.09 | <0.001 | 0.001 | <0.001 | <0.001 | <0.001 |
| ALP (U/L) | 456.00 | 145.00 | 299.00 | 317.00 | 338.00 | 381.00 | 23.14 | <0.001 | 0.001 | <0.001 | <0.001 | <0.001 |
| TC (mmol/L) | 2.06 | 1.23 | 1.67 | 1.87 | 1.77 | 1.89 | 0.08 | <0.001 | 0.005 | <0.001 | 0.001 | <0.001 |
| TG (mmol/L) | 0.50 | 0.53 | 0.54 | 0.55 | 0.56 | 0.54 | 0.03 | 0.804 | 0.926 | 0.876 | 0.804 | 0.950 |
| GLU (mmol/L) | 5.20 | 1.30 | 3.80 | 3.90 | 3.10 | 3.50 | 0.29 | <0.001 | 0.001 | <0.001 | <0.001 | <0.001 |
| Ca (mmol/L) | 2.51 | 2.20 | 2.30 | 2.48 | 2.42 | 2.48 | 0.04 | 0.021 | 0.390 | 0.034 | 0.087 | 0.034 |
| IP (mmol/L) | 3.11 | 2.08 | 2.44 | 2.95 | 2.69 | 2.83 | 0.11 | 0.003 | 0.221 | 0.008 | 0.047 | 0.019 |
| CRE (μmol/L) | 0.96 | 0.18 | 0.41 | 0.50 | 0.78 | 0.52 | 0.06 | <0.001 | 0.007 | 0.001 | <0.001 | <0.001 |
| HDL-C (mmol/L) | 1.12 | 0.45 | 0.83 | 0.97 | 0.87 | 1.04 | 0.06 | <0.001 | 0.001 | <0.001 | <0.001 | <0.001 |
| LDL-C (mmol/L) | 77.67 | 55.02 | 62.16 | 74.96 | 66.61 | 68.04 | 1.90 | <0.001 | 0.004 | <0.001 | <0.001 | <0.001 |
| UA (μmol/L) | 42.00 | 93.00 | 62.00 | 47.00 | 42.00 | 55.00 | 4.97 | <0.001 | 0.013 | 0.001 | <0.001 | 0.004 |
| γ-GT (U/L) | 4.00 | 11.50 | 4.80 | 4.13 | 3.17 | 4.50 | 0.71 | <0.001 | <0.001 | <0.001 | <0.001 | <0.001 |
| CK (U/L) | 1008.90 | 1354.80 | 1091.90 | 1034.20 | 1030.50 | 1031.20 | 38.16 | 0.005 | 0.024 | 0.008 | 0.008 | 0.008 |
