# Supplementary figures and images for: Baicalin and probenecid protect against Glaesserella parasuis challenge in a piglet model
Source: Vet Res. 2024 Jul 29;55:96. doi: 10.1186/s13567-024-01352-4 (PMC11285411; doi:10.1186/s13567-024-01352-4)

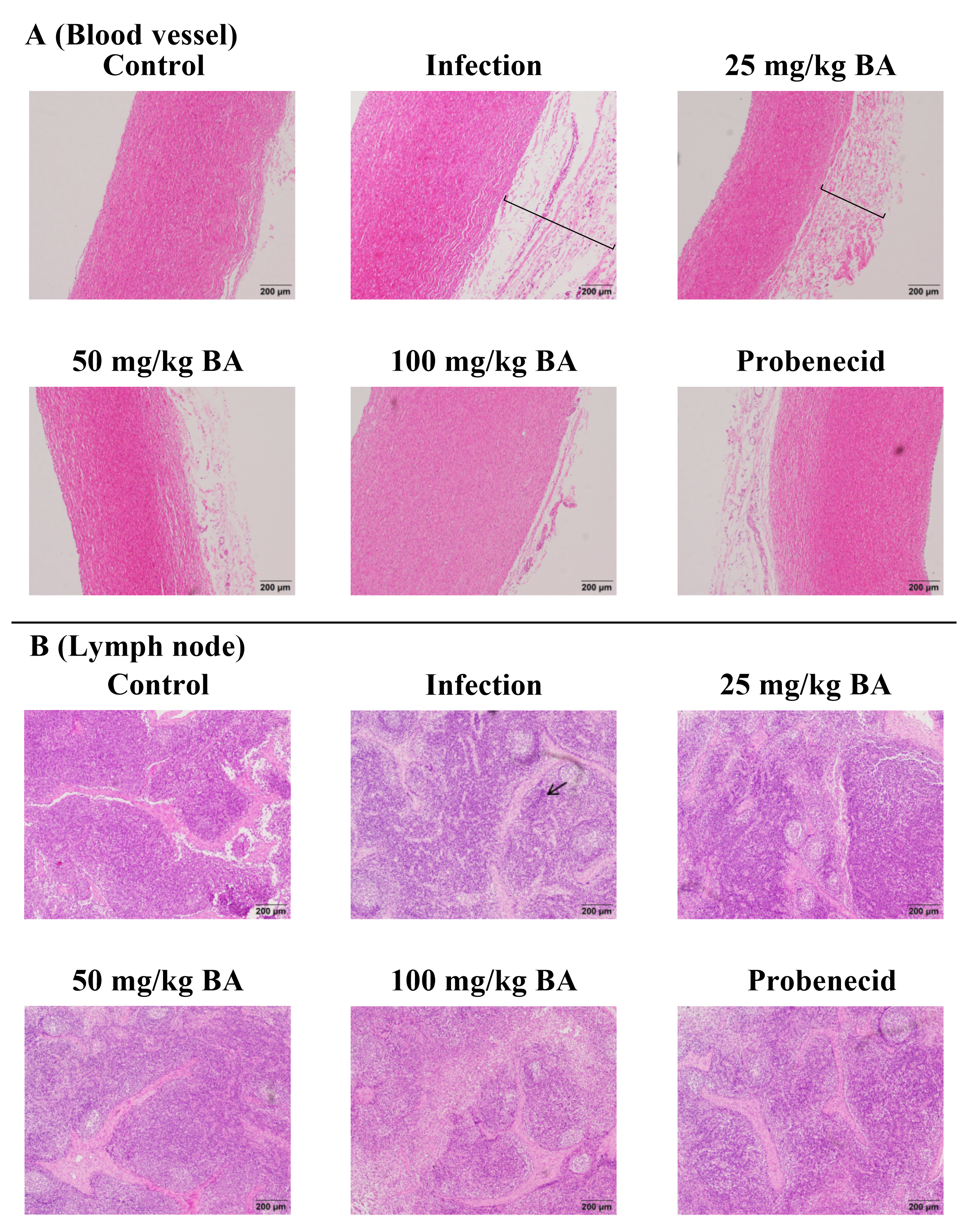

Supplement: Supplementary file 6 — Additional file 6. The effect of baicalin and probenecid on alleviating pathological tissue damage in piglets infected with G. parasuis. Blood vessels and lymph nodes were obtained to prepare tissue sections. A: Blood vessels; B: lymph node; BA: baicalin. [file 13567_2024_1352_MOESM6_ESM.tif]
